# The Impact of Body Mass Index on Local Anaesthetic Inguinal Hernia Repair

**DOI:** 10.7759/cureus.36163

**Published:** 2023-03-14

**Authors:** Mojolaoluwa Olugbemi, Thomas Athisayaraj, Emmanuel Lorejo, Eamonn Coveney

**Affiliations:** 1 General Surgery/Colorectal Surgery, West Suffolk Hospital, Bury St Edmunds, GBR; 2 General Surgery/Breast Surgery, West Suffolk Hospital, Bury St Edmunds, GBR

**Keywords:** unilateral, lichtenstein, bmi, local anaesthesia, inguinal hernia

## Abstract

Background: Open mesh repair of inguinal hernia is acceptable and can be performed under local anaesthesia (LA). Individuals with high BMI (Body Mass Index) have often been excluded from LA repairs for varying reasons including safety concerns. Open repair of unilateral inguinal hernia (UIH) amongst individuals with different BMI groups was studied. Its safety profile was investigated using LA volume and length of operation (LO) as endpoints. Operative pain and patient satisfaction were also evaluated.

Patients and methods: A total of 438 adult patients were studied having excluded underweight patients, those requiring any additional intra-operative analgesia, multiple procedures, or records with incomplete data. Operative pain, patient satisfaction, LO and LA volume were retrospectively studied from the existing data from clinical and operative notes.

Results: It was a predominantly male population (93.2% males) with an age range of 17-94 years peaking in the 60-69 years age group. BMI ranged 19-39 kg/m^2^ with BMI above normal at 62.8%. LO was 13-100 minutes (average 37 mins {SD = 12}) utilising an average LA volume of 45 ml (SD = 11) per patient. Across BMI groups, no significant difference in LO (P = 0.168) or patient satisfaction (P = 0.388) was seen. Although LA volume (P = 0.011) and pain score (P<0.001) demonstrated statistically significant differences, these did not appear to be clinically relevant. Over 90% in each BMI group experienced mild or no pain and with severe pain reported in only one patient in the entire population. Overall, LA volume required per patient was low and dosage was safe in all BMI groups with significant proportion (89%) of patients evaluated for satisfaction rating their experience ≥ 90 out of 100.

Conclusion: LA repair is safe and well tolerated irrespective of BMI. BMI is not a viable reason for exclusion of obese/overweight individuals from LA repair.

## Introduction

Body mass index (BMI) is a recognised means of comparing the weight and height of an individual serving as standard anthropometric tools [1]. Inguinal hernias (IH) account for three-quarters of anterior abdominal wall herniations [2,3] with their operative management evolving alongside surgery as a speciality [4]. Males are nine times more likely to develop a hernia in their lifetime, incidence also increases with age amongst adults [5,6]. The risk of developing a primary IH formation increases with a positive family history, abnormal collagen metabolism, and previous prostatectomy [4]. Obesity has been associated with a reduced risk of primary IH formation but an increased risk of a recurrent inguinal hernia after the repair of a primary hernia [7]. IH repairs are performed [3] using simple and complex operative approaches with no universally accepted method suitable in all patients [4]. The Lichtenstein repair [8,9] provides a uniform mesh-based approach with a favourable learning curve with easily reproducible results and significantly lower recurrence rates compared to its predecessors causing practice to move away from open suture/tension-based repairs [4,10]. This technique is still regarded as the “criterion standard” for an open repair approach when it is safe to use a mesh [4]. Individualisation of care demands tailoring the surgical approach to the surgeon, patient, hernia, clinical setting (emergency or elective) and available resources [6]. Open mesh repair remains relevant and, in some cases, the only feasible approach despite a recent surge in the use of laparoscopic approaches [4].

World Health Organisation (WHO) describes the “global epidemic of overweight and obesity as “globesity” with the UK experiencing an overall upward trend in the mean BMI in the last 25 years and the average resident being overweight [11-13]. Patients requiring IH repair increasingly include those with BMI above normal. These patients are likely also to have various obesity related medical problems, which though not directly related to their hernia, will have to be considered in the overall planning of their surgical care.

General anaesthesia (GA), regional anaesthesia (RA) and local anaesthesia (LA) with or without intravenous analgesia or sedation [9] are anaesthesia options for open IH repair [14-16]. LA is mostly used for elective procedures although its use in emergencies has also been reported [17]. Various LA preparations including buffered mixtures [18-20] as well as different approaches including local nerve blocks, field blocks, combination of local nerve blocks and field blocks amongst others are in use [21]. LA is the cheapest, simplest, safest and least invasive form of anaesthesia [22-25] allowing individuals with significant co-morbidities to be safely operated [23,26] facilitating day-case management of even high-risk patients. Inherent advantages in LA make it advantageous in obese patients who tend to have multiple co-morbidities and increased GA risks [27,28]. Safety in obese patients (LA volume) has been considered as a reason for excluding patients with high BMI [15] with clinicians considering problems that may arise with achieving adequate pain control within the safe limits of LA. Notably, LA is easily utilised even in low resource setting [25,29] allowing efficient use of scarce anaesthetic expertise [4]. The skills required to utilised LA is very minimal and many surgeons are comfortable to utilise LA both for field and nerve blocks. In the UK, LA repairs additionally improve the chance of meeting ≥75% day case rate for inguinal hernia repair [6,30].

The findings of this article were presented as a dissertation by the first author in the attainment of a ChM in General Surgery from the University of Edinburgh, College of Medicine and Veterinary Medicine in 2019. The findings of this article were presented as a poster at the 41st Annual EHS congress 2019, Hamburg, September 11th-14th, 2019.

## Materials and methods

We hypothesised that LA use in repair of unilateral inguinal hernias is safe and effective in overweight and obese patients. We studied the use of LA in open approach to treat inguinal hernias in normal, overweight and obese individuals evaluating operative pain score and patient satisfaction following LA inguinal hernia repair as well as safety of LA in different BMI groups (using the volume of local anaesthetic agent and length of operation as end points).

**Figure 1:**
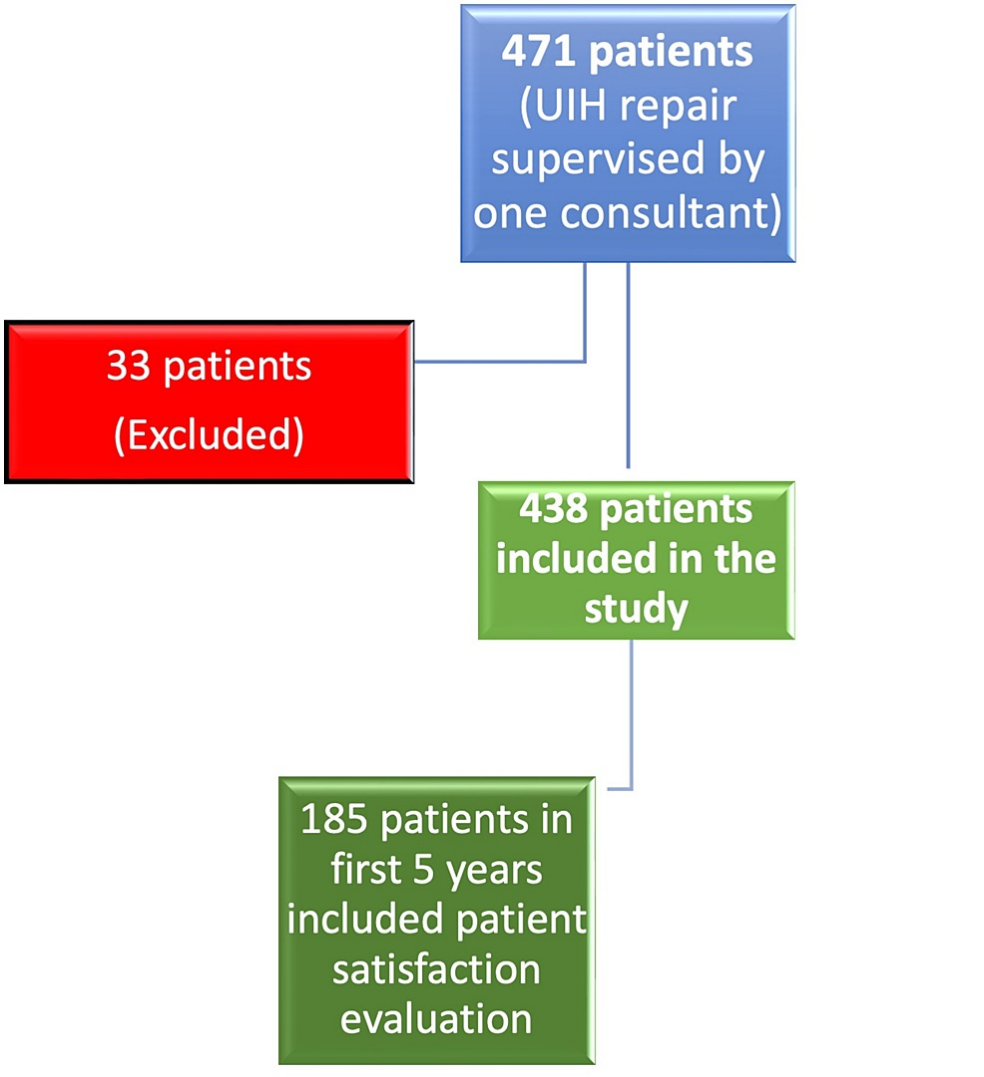
Patient flow chart

This was a retrospective study of patients who had open mesh repair of IH under LA supervised by a single consultant between 2010 and 2018. Permission to audit this data was sought and was obtained from the West Suffolk Hospital, Bury St Edmunds, UK and BMI Hospital, Bury St Edmunds, UK where all the procedures were performed. The clinical notes and operative records of patients who met the set criteria were reviewed. Of 471 consecutive patients identified (Figure 1), we included adults (≥ 16 years) who had open unilateral IH repair and excluded patients with incomplete data, underweight patients (BMI <18.5 Kg/m^2^) and those who required multiple procedures in the same sitting or any other form of analgesia intra-operatively. Furthermore, 185 patients audited in the first five years additionally had their satisfaction scores recorded within one hour of completing the procedure and this group had further evaluation of their patient satisfaction. Using anthropometric parameters (weight and height), Body Mass Index (BMI) was grouped based on the World Health Organisation (WHO) classification [1] into normal weight (BMI 18.5-24.9 Kg/m^2^), overweight (BMI 25-29.9 Kg/m^2^) or Obese (BMI ≥30 Kg/m^2^).

All the patients had elective open mesh repair (Lichtenstein repair). The procedures were performed using a standardised local anaesthesia mix constituted of 106 ml aliquots (20 ml 0.5% lidocaine with adrenaline, 30 ml of 0.5% bupivacaine with adrenaline, 50 ml of saline and 6 ml of 8.4% sodium bicarbonate). The operating surgeon performed preoperative field infiltration and ilioinguinal blockade using the buffered local anaesthetic solution. Each patient was provided with a dedicated team member who talked to them throughout the procedure, they engaged the patients in a positive way, provided attention control and avoided sympathising using negatively-loaded words as described by Lang et al. [31].

Data collection tools included patient records, theatre records, visual analogue pain scale, and visual analogue patient satisfaction scales aided by a data collection proforma. Information collected include patient demographics, volume of LA used, duration of procedure, duration of surgery, operative pain score (visual analogue scale {VAS}) and patient satisfaction proforma with the pain score and patient satisfaction scores collected as previously described. The subjective visual rating scale (VAS) was on a 100 mm long line anchored by two verbal descriptors at each extreme (no pain and severe pain). The subset of the patients who also had their satisfaction assessed at the same time utilised a visual scale (100 = very satisfied, 0 = dissatisfied).

Collected data was collated and analysed using IBM Statistical Package for Social Science (SPSS) for Windows, Version 24.0 (IBM Corp., Armonk, NY). Thirty-three patients were not included based on the exclusion criteria., of the 438 patients included, 185 patients were evaluated on their overall satisfaction during the audit. Descriptive statistics include the age (mean, standard deviation, age distribution across BMI groups), gender and BMI characteristics of the population. The pain score, length of operation (minutes), volume of LA used as well as the patient satisfaction scores were evaluated. The VAS score was interpreted as follows no pain (0-4 mm), mild pain (5-44 mm), moderate pain 45-74 mm) and severe pain (75-100 mm) [32,33]. Age was classified into three groups; young (<45 years), middle aged (45-64), elderly (>/=65 years). Normality of data was tested using Shapiro-Wilk test, the data distribution was skewed. The Kruskal-Wallis H test was used to evaluate significance across BMI groups. Correlations between BMI and volume of local anaesthetic agent used, operating time, pain score and patient satisfaction score were also evaluated. P value < 0.05 was considered significant. Amongst the 185 patients who provided patient satisfaction scores, the satisfaction score was compared with age and BMI categories.

## Results

This population was predominantly male with only 34 (7.8%) of the patients being females. They were aged 17-94 years with an average age of 64 years (SD = 14) and a peak in the 60-69 years age group. The mean BMI overall, was 25.9 kg/m^2^ (SD = 3.4) ranging 19 -39 kg/m^2^. Out of the total 50.2% of the participants were overweight, 12.6% were obese while the remaining 37.2% had a normal BMI. Majority of the young patients, one out of every two, had a normal BMI and only 4.4% of them being obese. Out of all elderly patients, 11.2% were obese and 46.4% were overweight. The distribution pattern amongst the middle-aged individuals differed with over half of them (57.4%) being overweight with this group additionally having the largest proportion of obese patients (16.6%).

The length of operation ranged between 13 and 100 minutes with an average of 37 minutes (SD = 12). Of the 106 ml aliquots of LA prepared for use for each patient, a minimum of 14 ml and a maximum of 90 ml were used to achieve anaesthesia with an average of 45 ml (SD = 11). Overall, the duration of the procedure did not significantly vary amongst the different BMI groups. This respectively averaged 36 mins, 38 mins, and 37 mins amongst normal, overweight and obese patients (P = 0.168). The overweight patients and obese patients averagely required 47 ml of LA for their procedure while individuals with normal BMI required less (43 ml). Although this was statistically significant (P = 0.011), the difference of 4 ml in the average volumes and the volumes being well within the safety limits of the LA renders this difference clinically irrelevant.

**Figure 2:**
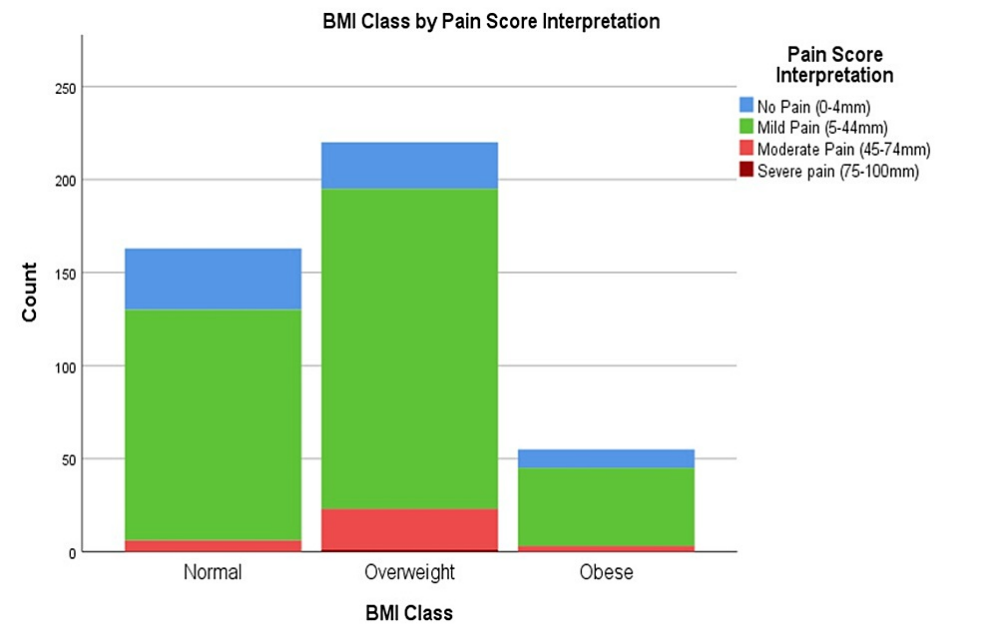
Pain scores across BMI groups BMI: Body Mass Index

Pain assessment with a Visual Analog Scale (VAS) was performed for all 438 participants. The mean VAS score was 18 (SD = 15), the lowest, 0 out of 100, was present in 9.8% of patients and the highest recorded pain score 80 was present in only one patient. 96.3% had VAS ≤50. Only 16 out of 438 patients had VAS above 50. On categorisation of the VAS, 15.5% had no pain, majority (77.2%) had mild pain, 7.1% had moderate pain and only 1 patient (0.2%) patient had severe pain. The average pain score was highest amongst the overweight individuals 21 (SD = 16) compared to obese individuals (18 SD = 16) and individuals with normal BMI (15 SD = 13). Although this difference in their pain score was statistically significant (P = <0.001), all the means were clinically within the mild pain category. Additionally, 89.5 % of these overweight individuals experienced either no pain or only mild pain. However, in comparison with other BMI categories, the overweight patients had the highest proportion of individuals in their group with moderate pain (10%) compared to 5.5% and 3.7% respectively amongst obese individuals and those with a normal BMI (Table 1, Figure 2).

**Table 1:** Local anaesthesia volume, procedure duration, patient satisfaction and pain scores across BMI groups BMI: Body Mass Index

Parameter	Normal BMI	Overweight	Obese
Mean volume of local anaesthetic agent used (ml) (P=0.011)	43 (SD = 12)	47 (SD = 11)	47 (SD = 12)
Mean duration of operation (mins) (P=0.168)	36 (SD = 10)	38 (SD = 12)	37 (SD = 12)
Mean patient satisfaction (scale 0-100) across different BMI categories (P=0.388)	96 (SD = 5)	94(SD = 11)	95(SD = 5)
VAS Pain Score distribution (%) within different BMI groups (P=0.005)			
No Pain (0-4 mm)	20.2	11.4	18.2
Mild Pain (5-44 mm)	76.1	78.2	76.4
Moderate Pain (45-74 mm)	3.7	10.0	5.5
Severe pain (75-100 mm)	-	0.5	-

**Figure 3:**
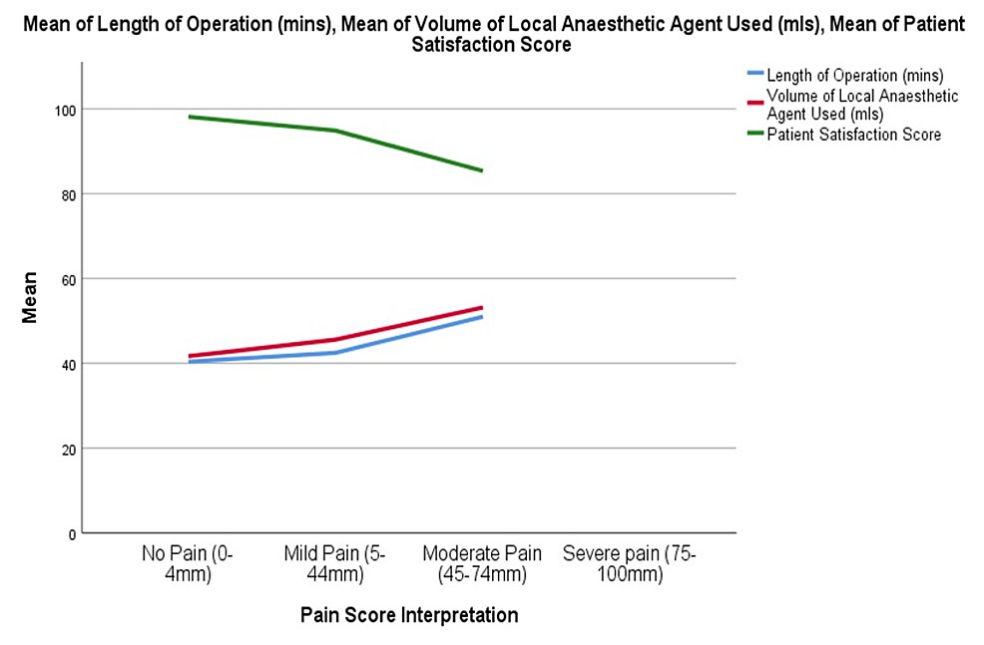
Changes in length of operation, volume of local anaesthetic agent (LA) and patient satisfaction in different pain score categories

Mean volume of LA (ml) required increased across pain categories 42 ml, 46 ml, 52 ml, and 58 ml progressing from no pain to severe pain (P<0.001). Furthermore, with ascending pain category, length of operation increased while patient satisfaction diminished (Figure 3). Of the 185 patients evaluated for their overall satisfaction, the mean score was 95 (SD = 9) out of a total of 100, while one patient rated lowest satisfaction score of 12 the scores of the other patients ranged 55-100. About 9 out of every 10 patients rated their satisfaction ≥ 90 irrespective of their BMI class with 25% of the patients rating their satisfaction as 100 and another 64% rating their satisfaction between 90 and 99. There were no statistically significant differences in the patient satisfaction score across different BMI categories (P respectively 0.388).

## Discussion

Open IH repairs are recognised as a safe and effective approach to repairing inguinal hernias. Male preponderance demonstrated (92.2%) is similar to most reports reflecting the significantly higher lifetime risk of this condition amongst males [3,34]. The peak age group in this study was 60-69 years The occurrence of IH is known to rise with increasing age amongst the adult population [7,35] with a recent mice study [36] suggesting age-related oestrogen increase contributes to this.

The mean BMI in this study population is within the overweight category with about two-thirds being obese or overweight especially middle-aged individuals [11,12,37]. Reid et al. [28] similarly observed that of their 125 patient hernia population, majority (72%) were overweight or obese. A raised BMI is associated with challenges in surgical care that occur both directly and indirectly because of the elevated BMI. These can result in significant problems in the overall care of these patients with regards to anaesthesia as well as the surgical procedure itself that can lead to a threat to life from a relatively minor procedure. Obese patients are more likely to experience adverse airway events [38] and other unfavourable outcomes following GA and benefit from a less invasive form of anaesthesia.

In most instances, inguinal hernia repairs last less than an hour avoiding patient discomfort on the operating table that can occur with a long operative procedure in awake patients. Here, the procedure averagely lasted 37 minutes and the longest lasted 100 minutes. The short duration allows most patients to remain comfortable and lie relatively still throughout the operation with adequate coverage by the duration of action of the LA. Sanjay and Woodward [16] using a similar LA mixture averagely required 60 ml/procedure which was more than was required (45 ml) by patients in this study with the highest volume utilised in a patient being 90 ml. Maximum safety concentration was therefore not compromised in all the patients [39]. In this study, LA is seen to be safe based on the set end points of volume of LA required to achieve anaesthesia during the procedure as well as the duration of the procedure itself, the latter being considered relative to the duration of action of the lidocaine and bupivacaine in the LA mix. Furthermore, although individuals with normal BMI required slightly less LA volume compared to overweight and obese patients, this was statistically significant but not clinically relevant as it was only a difference of 4 ml. Also, the duration of the procedure did not significantly vary with BMI. Overall, LA remained safe in all BMI groups [1,14].

Majority of the patients (92.7%) experienced either mild pain or no pain at all throughout their procedure and no patient experienced severe pain. There was no administration of any systemic analgesic and no conversion to general anaesthesia in all the patients. Lang et al. [31] studied the role of communication between a patient and healthcare staff. They observed amplified anxiety and distress when warnings and commiserations were utilised with structured attention improving their pain perception during the procedure. This guided our approach to the assignment of a team member to the patient during the procedure. This may contribute to our low overall pain scores and lower volume of anaesthetic solution used. Overweight and obese individuals had averagely higher VAS pain scores compared to individuals with normal BMI and this was statistically significant (p = 0.005). However clinically, this difference does not appear to be significant with the mean scores in each group remaining in the mild pain category irrespective of the BMI with similar pain control achieved in all the different BMI categories. The highest category of documented pain (moderate pain) was seen to occur more frequently in individuals who were middle aged or overweight. Nevertheless, it was well tolerated by all the age groups including in elderly patients. LA procedures can result in a high level of patient satisfaction. In this study, most of the patients were very satisfied with the procedure with 89% of them rating their experience 90 and above out of 100 irrespective of age or BMI. Often, patients who undergo LA procedures express satisfaction at the minimal interruption of their overall state; they can ambulate early and generally feel in control of their experience [40]. Nevertheless, we observed that as pain category progressed, length of operation and volume of LA required increased and patients were less satisfied with their procedure.

Patients with BMI above normal should not be denied LA repairs because of potential safety issues that may arise with required dosage necessary for satisfactory intra-operative pain control. The administration of LA requires minimal resources and can easily be fitted into already available facilities available to an institution offering minor and intermediate surgical procedures as specialised equipment is not required. It is easily a surgeon-led procedure that avoids the need for an anaesthetist [41] while providing results comparable to laparoscopic approaches with more readily reproducible outcomes and favourable learning curve [4,42]. Despite the relative ease of deploying LA services compared to a GA services, quality care requirements should not be compromised so outcomes can remain optimal.

Limitations

This was a retrospective review which was dependent on existing data and therefore satisfaction scores were only evaluated amongst patients treated in the first five years of the study period at which time satisfaction scores were also recorded. This study is at risk of selection bias which may have occurred during the booking of the inguinal hernia patients for local anaesthesia approach. Also, pain and satisfaction assessment tools were subjective parameters. This study only evaluated intra-operative use of local anaesthesia without documenting early and late postoperative complications.

## Conclusions

Day case procedures under LA are an invaluable tool in the care of symptomatic hernias both in high and low risk patients. It is a strategic tool in the repertoire of a complete hernia service, significantly improving costs allowing provision of services without specialised anaesthetic care in most instances, also resulting in improved day-case rates of IH. Pain control was similar irrespective of a BMI and age, and the operating time and volume of LA used did not significantly vary with the patient’s BMI. BMI should not be the sole reason for not offering or administering LA for open inguinal hernia repair to a patient requiring a unilateral open inguinal hernia procedure. Team members should be made aware of their ability to significantly impact outcomes by their choice of words and body language. Despite the presence of other treatment approaches, including GA-based repairs and laparoscopic repairs in the institutions of this study, LA hernia service remains a robust service in use in emergency and elective settings.
